# Androgen Deprivation Therapy–Induced Muscle Loss and Fat Gain Predict Cardiovascular Events in Prostate Cancer Patients

**DOI:** 10.1002/jcsm.13844

**Published:** 2025-06-04

**Authors:** Jie Lee, Yi‐Hsuan Lin, Pai‐Kai Chiang, Jhen‐Bin Lin, Ya‐Ting Jan, Wei‐Kung Tsai, Yu‐Jen Chen, Kun‐Pin Wu

**Affiliations:** ^1^ Department of Radiation Oncology MacKay Memorial Hospital Taipei Taiwan; ^2^ Department of Medicine MacKay Medical College New Taipei City Taiwan; ^3^ Institute of Biomedical Informatics National Yang Ming Chiao Tung University Taipei Taiwan; ^4^ Department of Urology MacKay Memorial Hospital Taipei Taiwan; ^5^ MacKay Junior College of Medicine, Nursing, and Management Taipei Taiwan; ^6^ Department of Radiation Oncology Changhua Christian Hospital Changhua Taiwan; ^7^ Department of Radiology MacKay Memorial Hospital Taipei Taiwan; ^8^ PhD Program of Interdisciplinary Medicine National Yang Ming Chiao Tung University Taipei Taiwan

**Keywords:** androgen deprivation therapy, body composition, cardio‐oncology, cardiovascular disease, prostate cancer

## Abstract

**Background:**

Androgen deprivation therapy (ADT) increases the risk of adverse cardiovascular events in patients with prostate cancer. ADT can induce body composition change; however, the association between body composition change and cardiovascular outcomes remains unclear. This study aimed to determine the association between ADT‐induced body composition change and cardiovascular outcomes in patients with prostate cancer.

**Methods:**

This retrospective study included 681 patients with prostate cancer (410 in the derivation cohort and 271 in the external validation cohort) who underwent radiotherapy and ADT between 2008 and 2021. Computed tomography (CT) scans at baseline and 6 months post‐ADT were used to assess the changes in skeletal muscle index (ΔSMI), subcutaneous adipose tissue index (ΔSATI) and visceral adipose tissue index (ΔVATI) at the L3 vertebral level. The primary outcome was major adverse cardiovascular events (MACEs), defined as a composite of myocardial infarction, stroke, heart failure, arterial revascularization and cardiovascular death. The association between body composition changes and MACE was analysed using the SHapley Additive ExPlanations (SHAP) method and validated using the Cox proportional hazards model.

**Results:**

The median age was 72 years (interquartile range: 66–77). With a median follow‐up of 6.0 years (derivation cohort) and 6.6 years (validation cohort), 62 (15.1%) and 39 (14.4%) patients experienced MACE, respectively. Six months post‐ADT, there was a reduction in SMI, whereas SATI and VATI had increased (*p* < 0.001). The ΔSMI and ΔSATI were the most important features for predicting MACE in both cohorts, whereas ΔVATI and baseline body composition parameters were less influential. SMI loss was inversely correlated with MACE risk, whereas increases in SATI and VATI were positively correlated. The identified thresholds for MACE prediction were SMI loss ≥ 4.7% and SATI gain ≥ 8.2%. On multivariable Cox regression analysis, ΔSMI (hazard ratio: 1.27 per 1% decrease, *p* < 0.001) and ΔSATI (hazard ratio: 1.07 per 1% increase, *p* < 0.001) were independently associated with MACE risk; however, ΔVATI was not (hazard ratio: 0.99, *p* = 0.23). Stratification based on thresholds confirmed that SMI loss ≥ 4.7% (hazard ratio: 10.58, *p* < 0.001) and SATI gain ≥ 8.2% (hazard ratio: 3.03, *p* < 0.001) were independently associated with increased MACE risk.

**Conclusions:**

ADT‐induced muscle loss and increased subcutaneous adipose tissue were associated with an increased MACE risk in patients with prostate cancer. These findings highlight the need to monitor and address body composition changes in patients undergoing ADT to reduce cardiovascular risk. Future research should explore interventions to mitigate these metabolic effects and improve patient outcomes.

**Abbreviations**
ADT: androgen deprivation therapy
ARSI: androgen receptor signalling inhibitors
AUC: area under the curve
BMI: body mass index
CatBoost: Categorical Boosting
CCI: Charlson Comorbidity Index
CI: confidence interval
CT: computed tomography
HR: hazard ratio
HU: Hounsfield unit
IQR: interquartile range
LHRH: luteinizing hormone‐releasing hormone
MACE: major adverse cardiovascular event
ML: machine learning
NCCN: National Comprehensive Cancer Network
PSA: prostate‐specific antigen
RF: random forest
SATI: subcutaneous adipose tissue index
SHAP: SHapley Additive exPlanations
SMI: skeletal muscle index
VATI: visceral adipose tissue index
XAI: explainable artificial intelligence
XGBoost: eXtreme Gradient Boosting

## 1 Introduction

Prostate cancer is the second most common cancer among men worldwide, with approximately 1 466 680 new cases reported in 2022 [1]. Due to advances in treatment and the indolent nature of the disease, cardiovascular disease has surpassed prostate cancer as the leading cause of mortality in these patients [2, 3, 4]. Many patients with prostate cancer present with multiple cardiovascular risk factors at diagnosis [5]. Therefore, identifying modifiable risk factors for cardiovascular disease in this population is essential [6].

Androgen deprivation therapy (ADT) is the standard systemic treatment for advanced‐stage prostate cancer and improves cancer‐specific survival. However, ADT is associated with an increased risk of major adverse cardiovascular events (MACEs), including myocardial infarction, stroke and heart failure [7]. The cardiovascular effects of ADT are largely mediated by metabolic alterations, including dyslipidaemia, insulin resistance and increased adiposity [8]. Notably, ADT induces significant changes in body composition, leading to muscle loss and increased adipose tissue accumulation [9, 10, 11, 12]. Skeletal muscle and adipose tissue are endocrine organs that regulate lipid metabolism, glucose homeostasis and inflammation through the production of myokines and adipokines [13, 14, 15]. Therefore, body composition is a potential target to reduce adverse cardiovascular outcomes in ADT‐treated patients [14]. One large prospective cohort study has linked higher baseline waist circumference and lower baseline muscle strength to an increased MACE risk in patients with prostate cancer [16]. However, the impact of ADT‐induced changes in muscle and fat on cardiovascular outcomes remains unclear. To address these gaps, studies are required to further evaluate the association between ADT‐induced changes in body composition with risk of MACE.

The relationship between body composition and cardiovascular disease is complex and likely nonlinear. Machine learning (ML) and explainable artificial intelligence (XAI) techniques allow for the modelling of nonlinear relationships and complex interactions [17, 18, 19]. The SHapley Additive exPlanations (SHAP) method can potentially provide an interpretable framework for understanding the relative contribution of body composition to MACE risk [20, 21, 22]. Regardless, scarce research has utilized ML and XAI to explore the relationship between body composition and cardiovascular outcomes in prostate cancer.

**FIGURE 1:**
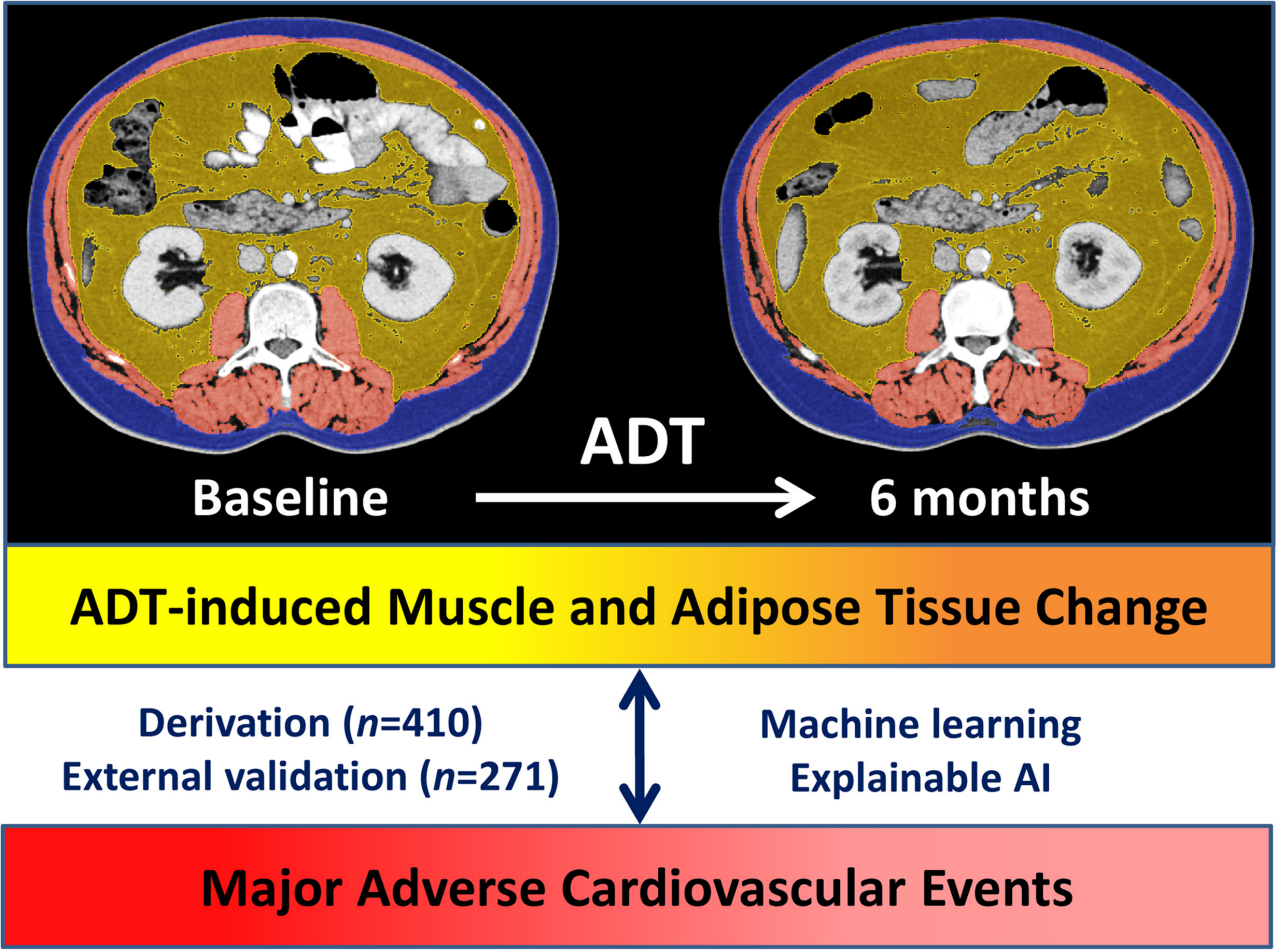
Utilization of explainable AI to explore the relationship of ADT‐induced body composition change with major adverse cardiovascular event. Body composition was assessed on a transversal computed tomography slice at the level of L3. ADT, androgen deprivation therapy; AI, artificial intelligence.

Given the unclear association between ADT‐induced body composition changes and cardiovascular disease, this study aimed to assess changes in muscle mass and adipose tissue using computed tomography (CT) imaging at baseline and 6 months after ADT initiation and to utilize ML and XAI to characterize the relationship between body composition changes and MACE risk in patients with prostate cancer (Figure 1).

## 2 Materials and Methods

### 2.1 Study Population

This retrospective cohort study was approved by the institutional review board of our institutions, which waived the requirement for informed consent due to its retrospective design. The study adhered to the Transparent Reporting of a multivariate prediction model for Individual Prognosis or Diagnosis (TRIPOD) guidelines [23]. Data from 838 patients diagnosed with National Comprehensive Cancer Network (NCCN) intermediate‐ or high‐risk prostate cancer who underwent radiotherapy and luteinizing hormone‐releasing hormone (LHRH) agonist at MacKay Memorial Hospital (derivation cohort, *n* = 507) or Changhua Christian Hospital (external validation cohort, *n* = 331) between 2008 and 2021 were reviewed. ADT duration was 6 months for intermediate‐risk prostate cancer and 2 years for high‐risk prostate cancer. Patients were excluded if they had (i) a history of malignancy, (ii) insufficient clinical information, (iii) missing follow‐up data, (iv) no CT scans at 6 months after ADT initiation or (v) inadequate quality of CT scans. Based on these criteria, 681 patients were included in the final analysis (derivation cohort: 410 patients; external validation cohort: 271 patients) (Figure S1).

### 2.2 Outcome Measures

The primary outcome measure was the first occurrence of a MACE, defined as a composite endpoint that included myocardial infarction, stroke, heart failure, arterial revascularization and cardiovascular death. The date and occurrence of MACE and the date of the last follow‐up were extracted from the institutional database. As patients could experience multiple cardiovascular events, only the time‐to‐first event was considered in the time‐to‐event model.

Baseline covariates, including age, Charlson Comorbidity Index (CCI), tumour stage, Gleason score, prostate‐specific antigen (PSA) level, smoking status and history of cardiovascular disease, diabetes and hypertension, were retrieved from institutional databases.

### 2.3 Body Composition Measurement

Skeletal muscle and adipose tissues were assessed using CT scans obtained at baseline and 6 months after ADT initiation. CT scans were performed with a consistent protocol, including contrast enhancement, 3‐mm slice thickness, 120 kVp and approximately 290 mA. A single researcher, blinded to patient information, measured the cross‐sectional areas (cm^2^) of skeletal muscle and adipose tissue on a single axial slice at the third lumbar vertebra (L3) level. Skeletal muscles include the psoas, rectus abdominis, paraspinal, transversus abdominis and internal and external oblique muscles. Subcutaneous and visceral adipose tissues were also assessed.

Body composition segmentation was performed using 3D Slicer software (version 4.11), applying predefined Hounsfield unit (HU) thresholds: −29 to +150 HU for skeletal muscle, −30 to −190 HU for subcutaneous adipose tissue and −50 to −150 HU for visceral adipose tissue [24, 25]. The cross‐sectional areas of the skeletal muscle and adipose tissues were normalized to the patient's height (cm^2^/m^2^) to calculate the skeletal muscle index (SMI), subcutaneous adipose tissue index (SATI) and visceral adipose tissue index (VATI). Body mass index (kg/m^2^) was collected within 1 week of each CT scan. Relative changes in SMI (ΔSMI), SATI (ΔSATI), VATI (ΔVATI) and BMI (ΔBMI) were calculated as follows:

$$\begin{array}{c} \text{Body composition change}\;\left(\%\right)\\ =\frac{{\text{Body composition}}_{\text{Second}\;\mathrm{CT}}-{\text{Body composition}}_{\text{First}\;\mathrm{CT}}}{{\text{Body composition}}_{\text{First}\;\mathrm{CT}}}\times 100 \end{array}$$

### 2.4 Machine Learning Models

The derivation cohort was used to train predictive models for MACE and characterize the relationships between body composition changes and MACE risk, while the external validation cohort was used to evaluate model performance and validate the relationships (Figure S1). Features included in the model were selected based on domain expertise and included age, CCI, tumour stage, Gleason score, PSA level, smoking status, history of cardiovascular disease, diabetes, hypertension and body composition parameters [3, 4, 5, 6, 8, 9, 10, 11, 12, 13, 14, 15, 16]. All features were standardized using *z*‐score normalization.

Random forest (RF), eXtreme Gradient Boosting (XGBoost) and Categorical Boosting (CatBoost) models were trained using the Python Scikit‐learn library (version 1.4.0; https://github.com/scikit‐learn/scikit‐learn.git), XGBoost (version 2.0.3; https://github.com/dmlc/xgboost.git) and CatBoost (version 1.2.2; https://github.com/catboost/catboost.git) [26]. In the derivation cohort, a random split was performed to create a training subset (70%) and a test subset (30%). Model training was conducted using 10‐fold cross‐validation with a grid search for hyperparameter tuning. To address the class imbalance in MACE outcomes, the borderline synthetic minority oversampling technique (borderline‐SMOTE) was applied. Model performance was evaluated using area under the curve (AUC), F1‐score, sensitivity, specificity and accuracy. These metrics were assessed using 500 bootstrap resamples from the derivation cohort, and results were reported with their 95% confidence intervals (CIs). The final model was selected based on performance in the validation cohort and subsequently analysed using SHAP to interpret the importance of body composition changes in MACE prediction.

### 2.5 SHAP Visualizations

SHAP values were calculated to evaluate the contribution of each input feature to MACE prediction. A SHAP summary plot was generated to illustrate feature importance within the model, while SHAP dependence plots were used to assess how individual features influenced model predictions. In the SHAP dependence plots, the actual feature values were plotted on the *x*‐axis, and the corresponding SHAP values were plotted on the *y*‐axis. The SHAP values for specific features exceeding zero push the decision towards the ‘MACE’ class, indicating a higher risk of MACE. According to, where the SHAP values exceed zero, the threshold of features associated with a higher risk of MACE can be determined [17, 18, 19, 20, 21]. The SHAP method was implemented using Python with SHAP version 0.40.0.

### 2.6 Statistical Analysis

Continuous variables were expressed as medians with interquartile ranges (IQRs) or mean with standard deviations, and comparisons were made using either the independent *t*‐test or the Mann–Whitney *U* test, as statistically appropriate. Categorical variables were reported as counts with percentages and analysed using the chi‐square test or Fisher's exact test, as appropriate. Changes in body composition were assessed using paired *t*‐test.

Following SHAP analysis, Kaplan–Meier estimates and Cox proportional hazard models were applied to confirm predictive associations. The Kaplan–Meier method was used to generate cumulative incidence curves for MACE, and the log‐rank test was conducted for comparisons between groups. Cox proportional hazard models were employed to estimate hazard ratios (HRs) and 95% CIs. Multivariable models were constructed using variables with *p* ≤ 0.1 in the univariable analysis. Due to potential multicollinearity between BMI and body composition parameters, these variables were analysed separately in multivariable models. All statistical analyses were performed using IBM SPSS software (version 21.0; IBM Corp., Armonk, NY, USA). A *p* value of < 0.05 was considered statistically significant.

## 3 Results

### 3.1 Patient Characteristics

In total, 681 patients were included, with a median age of 72 years (IQR, 66–77 years). Baseline patient characteristics, stratified by cohort, are presented in Table 1. No significant differences were observed between the derivation and external validation cohorts in age, CCI, tumour stage, Gleason score, PSA level, smoking status or history of cardiovascular disease, diabetes and hypertension. The median follow‐up was 6.0 years (IQR, 4.1–9.6 years) in the derivation cohort, during which 62 patients (15.1%) experienced MACE (myocardial infarction: 36; stroke: 10; heart failure: 14; revascularization: 44; cardiovascular death: 31). In the external validation cohort, the median follow‐up was 6.6 years (IQR, 4.4–11.2 years), with 39 patients (14.4%) experiencing MACE (myocardial infarction: 25; stroke: 6; heart failure: 7; revascularization: 27; cardiovascular death: 17).

**TABLE 1:** Patient and tumour characteristics.

Characteristics	Derivation cohort (*n* = 410)	External validation cohort (*n* = 271)	*p*
Age (years), median (IQR)	71 (66–77)	72 (66–76)	0.95
CCI, median (IQR)	4 (3–5)	4 (3–5)	0.90
T stage			0.98
T1	95 (23.2)	62 (22.9)	
T2	191 (46.6)	123 (45.4)	
T3	100 (24.4)	69 (25.5)	
T4	24 (5.9)	17 (6.3)	
Gleason score			0.55
6	74 (18.0)	48 (17.7)	
7	153 (37.3)	112 (41.3)	
8–10	183 (44.6)	111 (41.0)	
PSA level (ng/mL), median (IQR)	23.8 (14.7–44.4)	24.1 (16.1–44.4)	0.47
NCCN Risk group			0.18
Intermediate	80 (19.5)	42 (15.5)	
High	330 (80.5)	229 (84.5)	
Smoking	122 (29.8%)	81 (29.9%)	0.97
Cardiovascular disease	41 (10.0%)	29 (10.7%)	0.77
Diabetes	116 (28.3%)	80 (29.5%)	0.73
Hypertension	139 (33.9%)	95 (35.1%)	0.76
SMI (cm^2^/m^2^)			
Baseline	45.7 ± 7.6	45.4 ± 7.5	0.59
6‐month ADT	44.0 ± 7.7	43.9 ± 7.6	0.88
Change (%)	−3.8 ± 5.0	−3.4 ± 3.6	0.20
SATI (cm^2^/m^2^)			
Baseline	41.8 ± 14.6	41.7 ± 14.0	0.92
6‐month ADT	44.7 ± 15.5	44.1 ± 14.8	0.64
Change (%)	7.3 ± 8.7	6.1 ± 7.9	0.08
VATI (cm^2^/m^2^)			
Baseline	51.8 ± 26.6	51.5 ± 25.3	0.89
6‐month ADT	54.2 ± 27.8	53.8 ± 26.6	0.84
Change (%)	5.4 ± 10.3	4.7 ± 8.7	0.36
BMI (kg/m^2^)			
Baseline	24.3 ± 3.2	24.2 ± 3.2	0.69
6‐month ADT	24.9 ± 3.4	24.8 ± 3.4	0.75
Change (%)	2.2 ± 3.3	2.3 ± 2.9	0.75

*Note:* Data are mean ± standard deviation or number (%).

Abbreviations: ADT, androgen deprivation therapy; BMI, body mass index; CCI, Charlson Comorbidity Index; IQR, interquartile range; NCCN, National Comprehensive Cancer Network; SMI, skeletal muscle index; SATI, subcutaneous adipose tissue index; VATI, visceral adipose tissue index.

The median interval between CT scans was 182 days (IQR, 177–186 days) in the derivation cohort and 183 days (IQR, 177–187 days) in the validation cohort (*p* = 0.63). No significant differences in baseline body composition or changes in SMI, SATI, VATI and BMI were observed between the two cohorts (Table 1). After 6 months of ADT, patients in both cohorts exhibited a significant decrease in SMI and increases in SATI, VATI and BMI (*p* < 0.001).

### 3.2 Model Performance and Interpretation

In the derivation cohort, the RF, XGBoost and CatBoost models achieved AUCs of 0.910 (95% CI, 0.876–0.944), 0.884 (95% CI, 0.842–0.927) and 0.906 (95% CI, 0.872–0.940), respectively (Table S1, Figure S2). In the external validation cohort, the RF model outperformed the others, achieving an AUC of 0.920 (95% CI, 0.892–0.948), compared with 0.902 (95% CI, 0.862–0.941) for XGBoost and 0.916 (95% CI, 0.890–0.941) for CatBoost. Based on these results, the RF model was selected for SHAP analysis.

**FIGURE 2:**
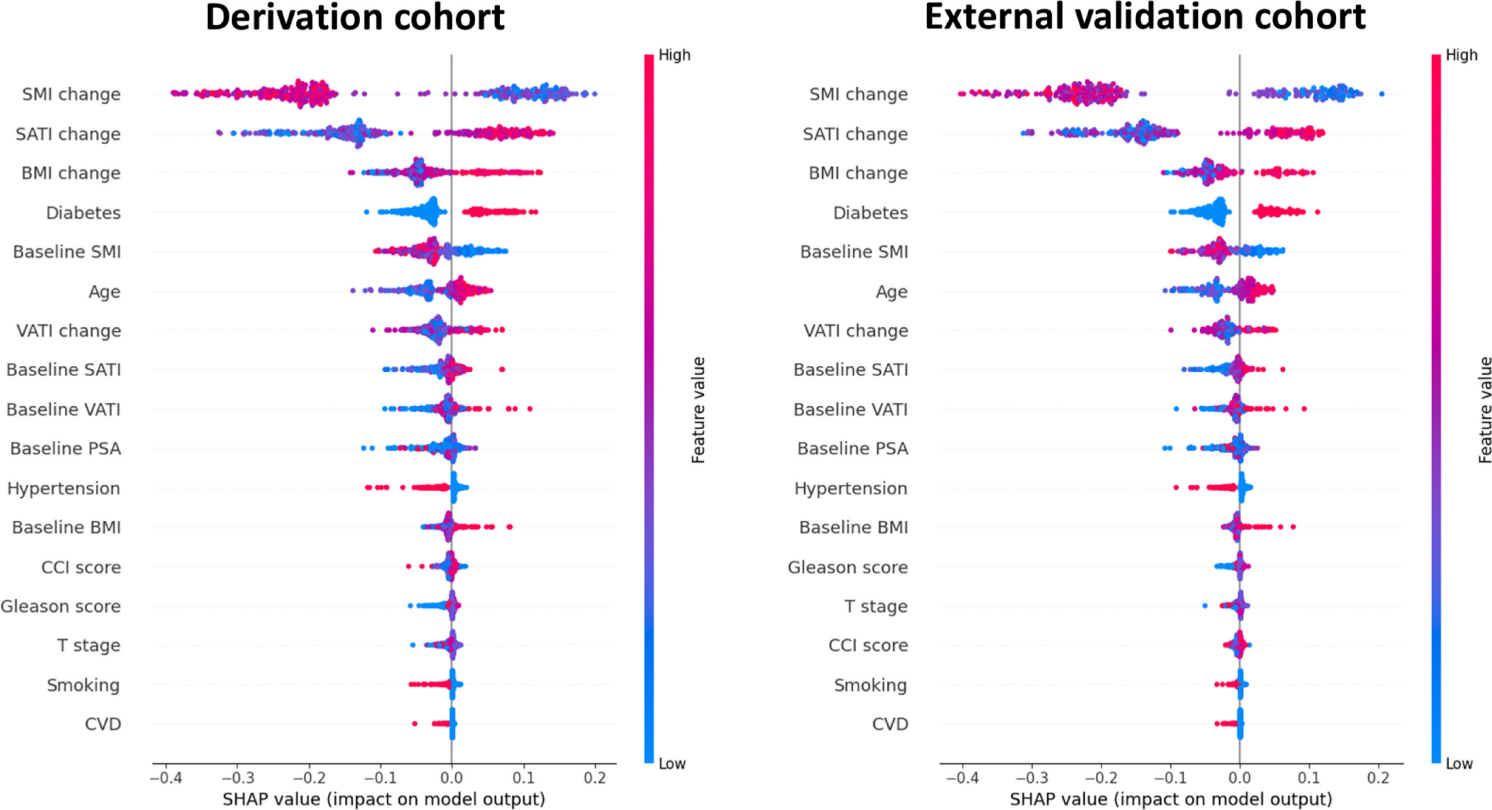
SHAP summary plots of derivation and external validation cohorts. SHAP summary plots rank the features in order of the global importance in the prediction. Each dot represents a SHAP value for a feature per patient, and red to blue colours represent the feature's value from high to low, respectively. SHAP, SHapley Additive ExPlanations.

**FIGURE 3:**
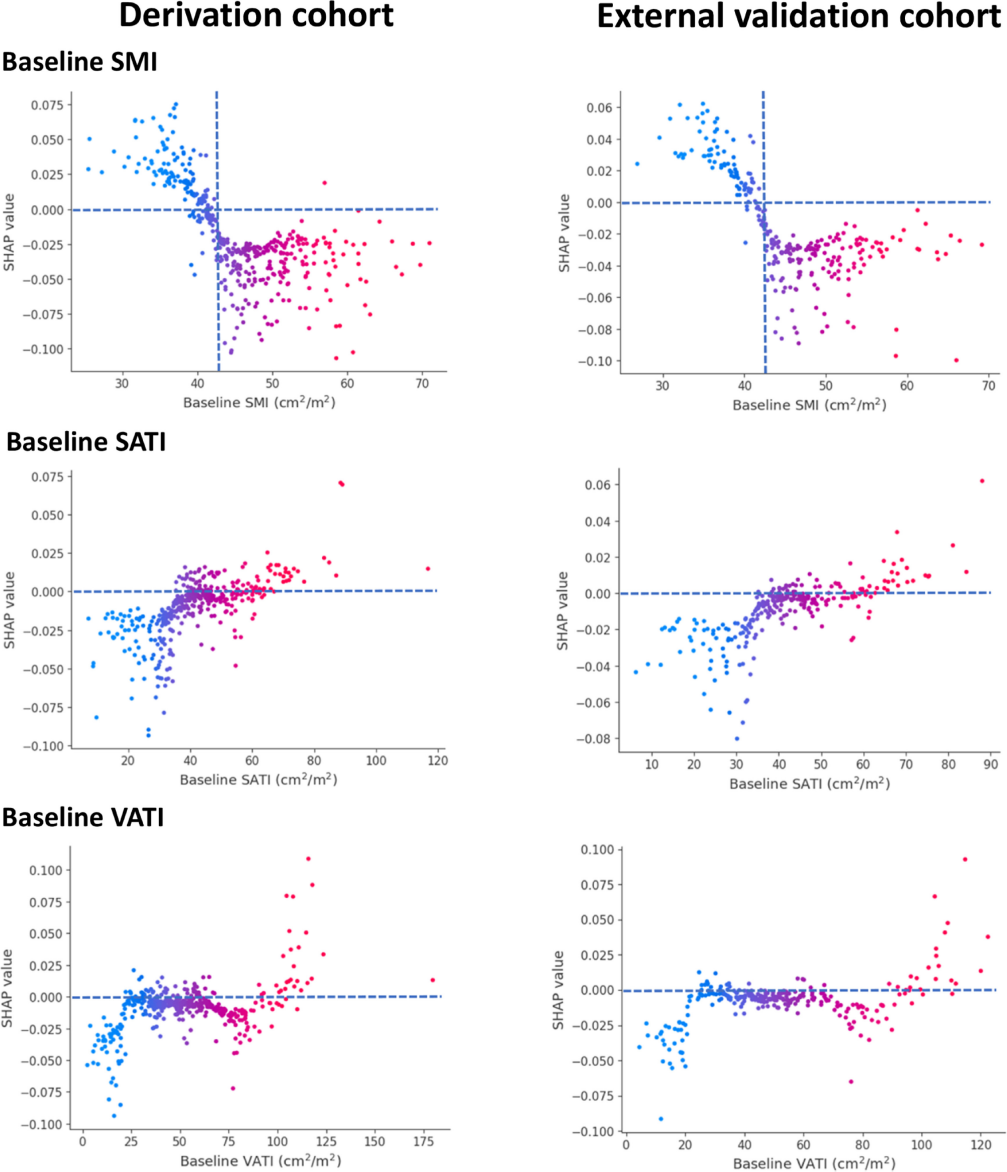
SHAP dependence plots of baseline body composition in the derivation and external validation cohorts. Each dot represents a SHAP value for a feature per patient, and red to blue colours represent the feature's value from high to low, respectively. SHAP values for specific features exceeding zero represent an increased risk of all‐cause mortality. SATI, subcutaneous adipose tissue index; SHAP, SHapley Additive ExPlanations; SMI, skeletal muscle index; VATI, visceral adipose tissue index.

**FIGURE 4:**
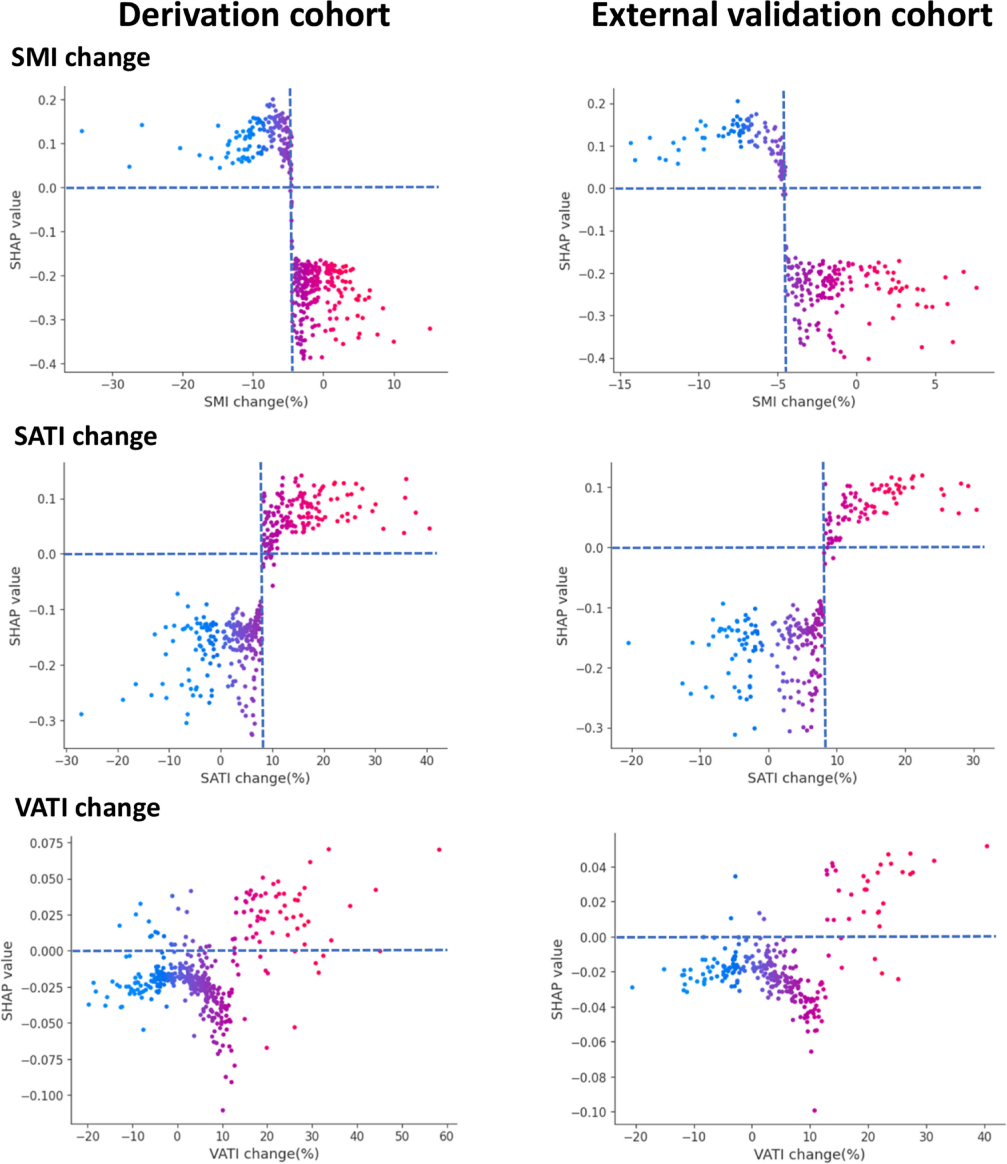
SHAP dependence plots of ADT‐induced body composition change in the derivation and external validation cohorts. Each dot represents a SHAP value for a feature per patient, and red to blue colours represent the feature's value from high to low, respectively. SHAP values for specific features exceeding zero represent an increased risk of all‐cause mortality. ADT, androgen deprivation therapy; SATI, subcutaneous adipose tissue index; SHAP, SHapley Additive ExPlanations; SMI, skeletal muscle index; VATI, visceral adipose tissue index.

**FIGURE 5:**
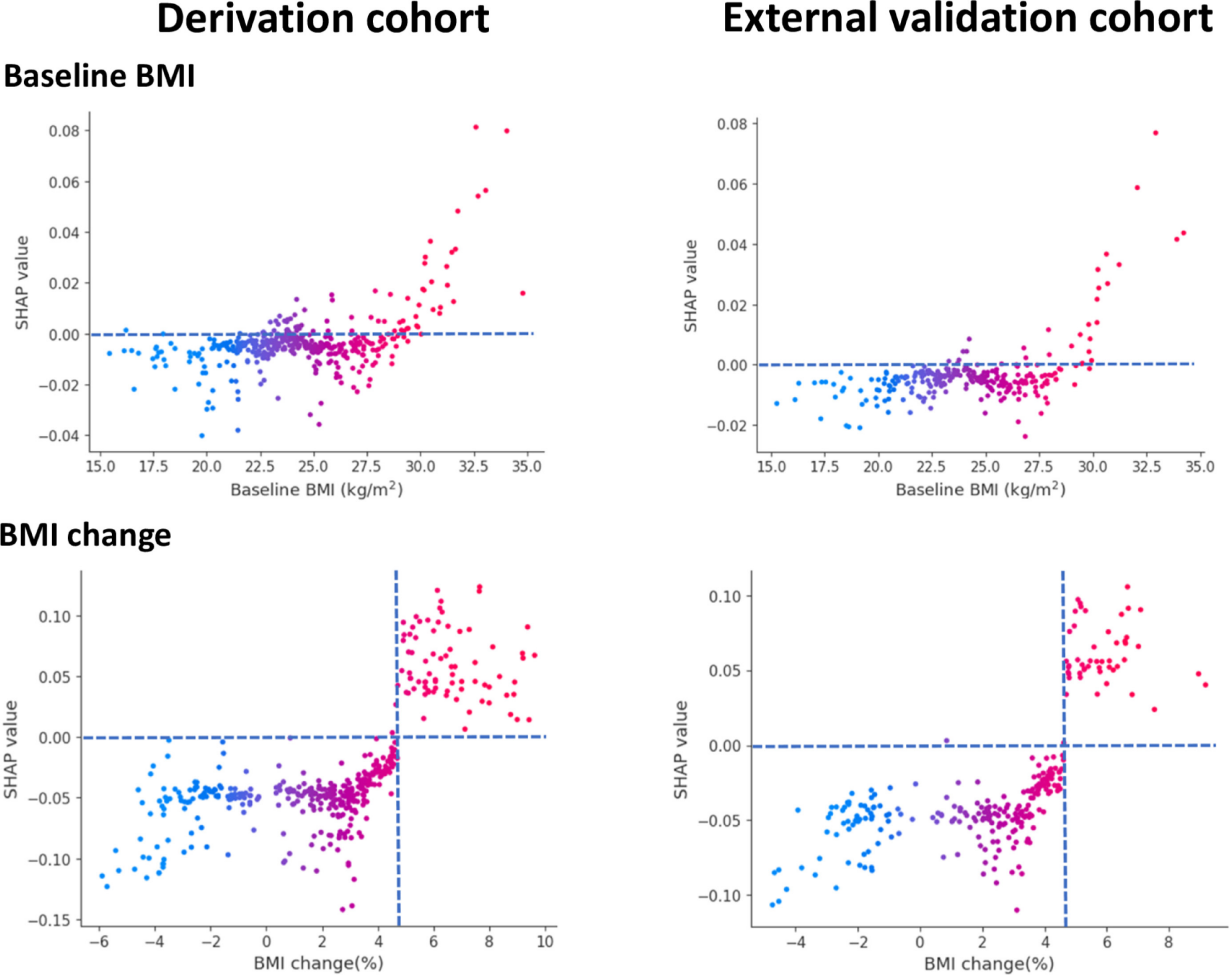
SHAP dependence plots of body mass index at baseline and change in the derivation and external validation cohorts. Each dot represents a SHAP value for a feature per patient, and red to blue colours represent the feature's value from high to low, respectively. SHAP values for specific features exceeding zero represent an increased risk of all‐cause mortality. BMI, body mass index; SHAP, SHapley Additive ExPlanations.

The SHAP summary plots provide a global interpretation of the model, ranking the importance of each feature in predicting MACE (Figure 2). Across both cohorts, ΔSMI, ΔSATI and ΔBMI emerged as the three most influential predictors, whereas ΔVATI and baseline body composition contributed less to the model's predictive performance. SHAP dependence plots revealed nonlinear relationships between body composition parameters and SHAP value. Baseline SMI exhibited an inverse correlation with SHAP value, while baseline SATI and VATI were positively correlated with SHAP values (Figure 3). Similarly, a decrease in ΔSMI was inversely correlated with SHAP values, whereas increases in ΔSATI and ΔVATI were positively correlated with SHAP values (Figure 4). The baseline BMI and ΔBMI were positively correlated with the SHAP value (Figure 5). These findings suggest that a decline in SMI or an increase in SATI, VATI or BMI–either at baseline or after 6 months of ADT–is associated with a higher risk of MACE.

In both cohorts, baseline SMI exceeded the SHAP threshold of 0 at approximately 42.8 cm^2^/m^2^, potentially serving as a cut‐off for defining baseline sarcopenia. SHAP analysis further identified risk thresholds for changes in body composition, where SMI loss ≥ 4.7%, SATI gain ≥ 8.2% and BMI gain ≥ 4.7% were associated with an elevated MACE risk. However, the distributions of baseline SATI, VATI, BMI and ΔVATI showed considerable overlap around the SHAP threshold of 0, preventing the determination of clear predictive cut‐offs for these parameters.

Based on these SHAP‐derived thresholds, 258 patients (37.9%) in the overall cohort had baseline sarcopenia. After 6 months of ADT, 239 (35.1%), 259 (38.0%) and 126 (18.5%) patients had SMI loss ≥ 4.7%, SATI gain ≥ 8.2% and BMI gain ≥ 4.7%, respectively. No statistically significant differences in body composition changes were observed between patients with and without baseline sarcopenia (Table S2).

### 3.3 Cox Regression

**FIGURE 6:**
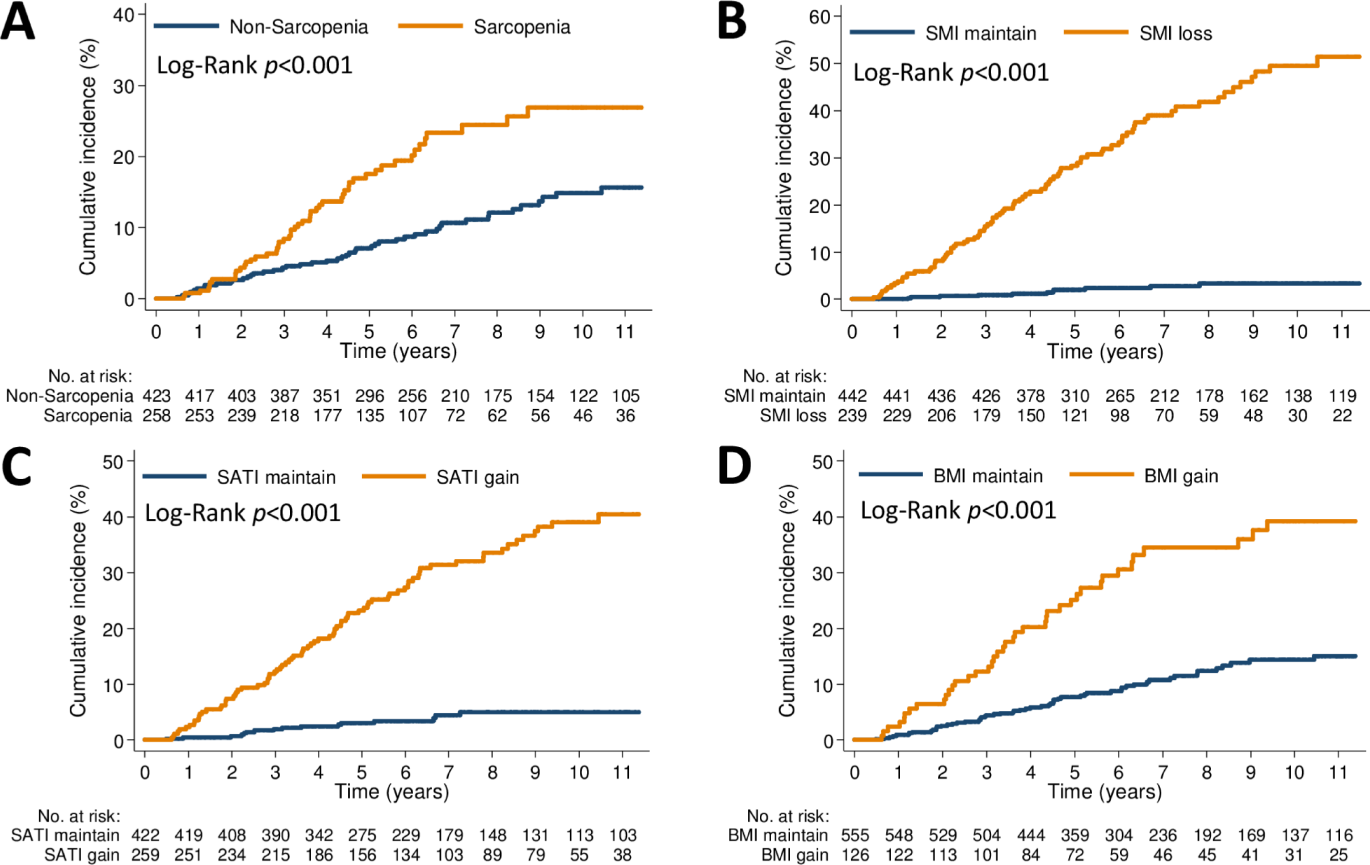
Kaplan–Meier curves for incident major adverse cardiovascular events stratified by the threshold of baseline SMI (A), SMI change (B), SATI change (C) and BMI change (D) in the overall cohort. BMI, body mass index; SATI, subcutaneous adipose tissue index; SMI, skeletal muscle index.

Baseline sarcopenia, SMI loss ≥ 4.7%, SATI gain ≥ 8.2% and BMI gain ≥ 4.7% were significantly associated with an increased risk of MACE (Figure 6). In univariable analysis, age, CCI, smoking, history of cardiovascular disease, diabetes, baseline SMI, ΔSMI, ΔSATI, ΔVATI and ΔBMI were associated with MACE risk (Table S3). However, baseline SATI, VATI, BMI, NCCN risk group and hypertension were not significantly associated with MACE risk. In the multivariable analysis, body composition parameters were analysed as both continuous and categorical variables (Table 2). In model A (continuous variables), baseline SMI, ΔSMI and ΔSATI were independently associated with an increased MACE risk, whereas ΔVATI was not. In model B (categorical variables), SMI loss ≥ 4.7%, SATI gain ≥ 8.2% and ΔVATI were independently associated with an increased MACE risk. However, baseline sarcopenia was not an independent predictor of MACE. Additionally, ΔBMI or BMI gain ≥ 4.7% was independently associated with an increased MACE risk.

**TABLE 2:** Multivariable Cox proportional hazards model for major adverse cardiovascular events in the overall cohort.

Variable	HR (95% CI)^a^, ^b^	*p* value
Model A		
Baseline SMI (1 cm^2^/m^2^ decrease)	1.03 (1.01–1.06)	0.02
ΔSMI (per 1% decrease)	1.27 (1.21–1.32)	< 0.001
ΔSATI (per 1% increase)	1.07 (1.05–1.10)	< 0.001
ΔVATI (per 1% increase)	0.99 (0.96–1.01)	0.23
Model B		
Baseline sarcopenia^c^	1.49 (0.98–2.25)	0.06
SMI loss ≥ 4.7% (vs. SMI maintain)	10.58 (5.55–20.15)	< 0.001
SATI gain ≥ 8.2% (reference: SATI maintain)	3.03 (1.68–5.46)	< 0.001
ΔVATI (per 1% increase)^d^	1.02 (1.01–1.04)	0.03
Model C		
ΔBMI (per 1% increase)	1.19 (1.10–1.29)	< 0.001
Model D		
BMI gain ≥ 4.7% (vs. BMI maintain)^b^	3.71 (2.47–5.59)	< 0.001

Abbreviations: BMI, body mass index; CI, confidence interval; HR, hazard ratio; SMI, skeletal muscle index; SATI, subcutaneous adipose tissue index; VATI, visceral adipose tissue index.

^a^ Adjusted for age, Charlson Comorbidity Index, smoking, history of cardiovascular disease and diabetes.

^b^ Baseline SATI, VATI and BMI were not analysed in the multivariable model because these parameters were not associated with MACE risk in univariable analysis.

^c^ SMI < 42.8 cm^2^/m^2^ was defined as sarcopenia.

^d^ ΔVATI was analysed as continuous variable because its threshold could not be identified based on SHAP analysis.

## 4 Discussion

To the best of our knowledge, this is the first study to characterize the relationship between ADT‐induced body composition changes and MACE risk in patients with prostate cancer. Our findings demonstrate that in ADT‐treated patients, the association between body composition and MACE is nonlinear, highlighting the utility of ML and XAI for risk prediction. Across both cohorts, ΔSMI, ΔSATI and ΔBMI were the most important features for predicting MACE risk. A decrease in SMI was inversely correlated with MACE risk, whereas increases in SATI, VATI and BMI were positively correlated with MACE risk. Thresholds for ΔSMI, ΔSATI and ΔBMI were identified and externally validated as predictors of MACE. These findings suggest that monitoring and managing body composition changes during ADT could be a strategy for reducing cardiovascular risk.

Body composition is a biomarker of overall health that provides prognostic information for men with prostate cancer [3]. In this study, it was found that patients had muscle loss and increased adipose tissue after 6 months of ADT. The observation is consistent with previous studies on the changes in body composition during ADT in prostate cancer [9, 10, 11]. Recently, Leong et al. revealed that lower baseline muscle strength and higher waist circumference are associated with an increased MACE risk in patients with prostate cancer [16]. However, there is a lack of analysis of the association of changes in muscle strength and waist circumference with cardiovascular outcomes in their study. Our study builds on this by further demonstrating that ADT‐induced muscle loss and subcutaneous adipose tissue gain were independently associated with MACE risk. Notably, the differences in measurements of muscle and fat between studies should be discussed. In their study, they measured muscle strength by handgrip and quantified fat by waist circumference, whereas we quantified muscle and adipose tissue on CT scans. The waist circumference and CT‐based adipose tissue measurement are both to quantify fat and may have a similar prognostic role. However, muscle strength is a measure of muscle function, while CT‐based muscle measurement is a measure of muscle quantity [27]. Therefore, muscle strength and quantity may have different effects in predicting cardiovascular outcomes. Although ADT‐induced muscle loss was found to be associated with increased MACE risk, future study is required to evaluate the association between changes in handgrip strength during ADT and cardiovascular outcomes.

The use of XAI can rank the importance of factors to predict outcomes and visualize the inner workings of prediction in prostate cancer [17]. XAI‐based SHAP analysis was applied to demonstrate that changes in body composition were stronger predictors of MACE than baseline measures. Among these, muscle loss and subcutaneous adipose tissue gain also had greater importance than BMI changes, emphasizing the importance of monitoring body composition beyond traditional BMI measurements. Moreover, muscle loss was also found to be inversely correlated with MACE risk, whereas an increase in adipose tissue was positively correlated. These findings suggest that body composition is a target for lowering MACE risk and underscore the need for strategies aimed at preventing muscle loss and fat accumulation during ADT [14].

To prevent muscle loss and fat gain during ADT, several interventions, including exercise, diet/nutrition and multidisciplinary care involving cardio‐oncologists, can be considered. Supervised resistance, aerobic and impact exercises, along with prolonged exercise durations, can increase muscle mass and reduce fat accumulation in men undergoing ADT [28, 29, 30, 31]. Newton et al. revealed that exercise either at the onset or after 6 months of ADT can enhance muscle strength and physical function [29]. To avoid initial treatment‐related adverse effects, implementing exercise at ADT onset is suggested. Integrating exercise with diet/nutritional interventions may potentially enhance outcomes; however, studies reported that exercise‐only intervention was more effective in increasing lean mass and reducing body fat mass than exercise with diet/nutrition [28]. Although we suggest diet/nutrition may play a role in preventing muscle loss and fat gain during ADT, additional studies are needed to confirm their effectiveness. Moreover, patients with prostate cancer often have multiple cardiovascular risk factors [32]. It is essential to collaborate with cardio‐oncologists to regularly monitor blood pressure, lipid panels and fasting glucose levels and control modifiable cardiovascular factors for these patients [5, 6, 7, 33]. Based on the findings, initiating these interventions at ADT onset is suggested to prevent muscle loss and fat gain and potentially lower the cardiovascular risk.

The thresholds for baseline SMI, ΔSMI, ΔSATI and ΔBMI were identified using SHAP analysis. However, baseline sarcopenia was not independently associated with MACE risk in multivariable analysis, whereas SMI loss ≥ 4.7% remained a significant independent predictor of MACE. This suggests that muscle loss during ADT has a stronger impact on cardiovascular risk than baseline muscle mass. Therefore, the thresholds identified in this study may help guide enhanced interventions to improve considerable muscle loss and fat gain during ADT.

This study has several limitations. As a retrospective analysis, it is subject to potential confounding factors that may not have been fully accounted for. Additionally, key metabolic markers, including triglyceride and cholesterol levels, fasting glucose, muscle strength and dietary intake, were not available for most patients, limiting the ability to fully access metabolic mechanisms underlying ADT‐induced body composition changes and MACE risk. Another limitation is that this study focused solely on body composition changes after 6 months of ADT, preventing the evaluation of long‐term trends and their effects on cardiovascular outcomes. Future research should investigate whether continued muscle loss or fat gain beyond 6 months further exacerbates MACE risk. Moreover, this study examined patients treated with LHRH agonists, but androgen receptor signalling inhibitors (ARSI) may induce body composition changes that are distinct from those induced by LHRH agonists [34]. Further studies are needed to determine whether ARSI‐induced changes in body composition differentially influence cardiovascular risk.

In conclusion, this study provides evidence that ADT‐induced muscle loss and subcutaneous adipose tissue gain are associated with an increased MACE risk in patients with prostate cancer. Muscle loss and subcutaneous adipose tissue gain after 6 months of ADT were the most important predictors of MACE risk. These findings suggest that monitoring and managing body composition changes during ADT may help reduce cardiovascular risk. Further studies are required to determine whether strategies aimed at enhancing muscle mass and reducing adipose tissue accumulation could mitigate adverse cardiovascular outcomes in ADT‐treated patients. Incorporating these findings into clinical practice could establish proactive body composition management as a key component of cardiovascular risk reduction strategies in prostate cancer care.

## Ethics Statement

This study was conducted in accordance with the Declaration of Helsinki. This study was approved by the Institutional Review Boards of MacKay Memorial Hospital (24MMHIS392e) and Changhua Christian Hospital (240910).

## Conflicts of Interest

The authors declare no conflicts of interest.

## Declaration of Generative AI and AI‐Assisted Technologies in the Writing Process

The authors declare no Generative AI and AI‐assisted technologies in the writing process.

## Supporting information


**Table S1** Evaluation of model performance in the derivation and external validation cohorts.
**Table S2** Body composition changes according to pre‐treatment sarcopenia status.
**Table S3** Univariable Cox proportional hazards model for major adverse cardiovascular events.

**Figure S1.** Summary of overall analytical methodolody. CCH, Changhua Christian Hospital; CT, computed tomograpgy; MMH, MacKay Memorial Hospital.

**Figure S2.** The best performing receiver operating characteristic curves of the machine learning models.
