# Supplementary material for: Androgen Deprivation Therapy–Induced Muscle Loss and Fat Gain Predict Cardiovascular Events in Prostate Cancer Patients
Source: J Cachexia Sarcopenia Muscle. 2025 Jun 4;16(3):e13844. doi: 10.1002/jcsm.13844 (PMC12134783; doi:10.1002/jcsm.13844)
Supplement: Supplementary file 1 — Table S1 Evaluation of model performance in the derivation and external validation cohorts. Table S2 Body composition changes according to pre‐treatment sarcopenia status. Table S3 Univariable Cox proportional hazards model for major adverse cardiovascular events. [file JCSM-16-e13844-s003.docx]

**Supplementary Table 1** Evaluation of model performance in the derivation and external validation cohorts.

|  | **AUC** | **F1-score** | **Sensitivity** | **Specificity** | **Accuracy** |
| --- | --- | --- | --- | --- | --- |
| **Derivation cohort*** | | | | | |
| RF | 0.910 (0.876, 0.944) | 0.543 (0.392, 0.693) | 0.569 (0.346, 0.792) | 0.912 (0.866, 0.957) | 0.861 (0.819, 0.903) |
| XGBoost | 0.884 (0.842, 0.927) | 0.519 (0.384, 0.653) | 0.625 (0.424, 0.826) | 0.866 (0.815, 0.917) | 0.831 (0.781, 0.881) |
| CatBoost | 0.906 (0.872, 0.940) | 0.587 (0.465, 0.710) | 0.742 (0.519, 0.964) | 0.867 (0.824, 0.910) | 0.849 (0.809, 0.889) |
| **External validation cohort*** | | | | | |
| RF | 0.920 (0.892, 0.948) | 0.575 (0.472, 0.677) | 0.556 (0.391, 0.721) | 0.938 (0.907, 0.968) | 0.883 (0.859, 0.906) |
| XGBoost | 0.902 (0.862, 0.941) | 0.575 (0.483, 0.668) | 0.627 (0.476, 0.779) | 0.907 (0.865, 0.949) | 0.867 (0.834, 0.900) |
| CatBoost | 0.916 (0.890, 0.941) | 0.583 (0.491, 0.674) | 0.648 (0.485, 0.810) | 0.904 (0.866, 0.942) | 0.867 (0.838, 0.896) |

Abbreviations: AUC, area under the receiver operating characteristic curve; CatBoost, categorical boosting; RF, random forest; XGBoost, extreme gradient boosting.

* The performance of each model was calculated with 95% confidence interval (CI) generated using 500 bootstraps.

**Supplementary Table 2** Body composition changes according to pre-treatment sarcopenia status.

|  | **No baseline sarcopenia (*n*=423)** | **Baseline sarcopenia (*n*=258)** | ***P*** |
| --- | --- | --- | --- |
| **SMI change (%)** | -3.5 ± 4.6 | -3.9 ± 4.2 | 0.28 |
| **SATI change (%)** | 6.4 ± 8.5 | 7.5 ± 8.3 | 0.12 |
| **VATI change (%)** | 4.7 ± 9.4 | 5.7 ± 10.3 | 0.19 |
| **BMI change (%)** | 2.2 ± 3.1 | 2.2 ± 3.2 | 0.99 |

Abbreviations: BMI, body mass indes; SMI, skeletal muscle index; SATI, subcutaneous adipose tissue index; VATI, visceral adipose tissue index.

Data are Data are mean ± standard deviation.

**Supplementary Table 3** Univariable Cox proportional hazards model for major adverse cardiovascular events.

| **Variable** | **Hazard ratio (95% CI)** | ***P*** |
| --- | --- | --- |
| Age (years) | 1.05 (1.03-1.08) | 0.001 |
| CCI score, continuous | 1.50 (1.29-1.75) | <0.001 |
| NCCN high risk group (Reference: Intermediate) | 1.18 (0.69-2.01) | 0.55 |
| Smoking | 2.49 (1.68-3.68) | <0.001 |
| Cardiovascular disease | 1.82 (1.04-3.21) | 0.04 |
| Diabetes | 3.51 (2.37-5.20) | <0.001 |
| Hypertension | 1.15 (0.77-1.72) | 0.50 |
| Baseline SMI (1 cm^2^/m^2^ decrease) | 1.03 (1.01-1.06) | 0.02 |
| Baseline Sarcopenia^b^ | 2.12 (1.43-3.13) | <0.001 |
| ΔSMI (per 1% decrease) | 1.25 (1.21-1.29) | <0.001 |
| SMI loss ≥4.7 % (vs. SMI maintain)^b^ | 19.02 (10.38-34.84) | <0.001 |
| Baseline SATI (1 cm^2^/m^2^ increase) | 1.01 (0.99-1.02) | 0.41 |
| ΔSATI (per 1% increase) | 1.09 (1.07-1.11) | <0.001 |
| SATI gain ≥8.2 % (Reference: SATI maintain)^b^ | 9.62 (5.63-16.42) | <0.001 |
| Baseline VATI (1 cm^2^/m^2^ increase) | 1.00 (0.99-1.01) | 0.33 |
| ΔVATI (per 1% increase) | 1.05 (1.03-1.07) | <0.001 |
| Baseline BMI (1 kg/m^2^ increase) | 0.96 (0.91-1.02) | 0.22 |
| ΔBMI (per 1% increase) | 1.21 (1.12-1.30) | <0.001 |
| BMI gain ≥4.7 % (vs. BMI maintain)^b^ | 3.61 (2.43-5.35) | <0.001 |

Abbreviations: BMI, body mass index; CI, confidence interval; CCI, Charlson Comorbidity Index; SMI, skeletal muscle index; SATI, subcutaneous adipose tissue index; VATI, visceral adipose tissue index.

^a^ SMI <42.8 cm^2^/m^2^ was defined as sarcopenia.
