# Supplementary figures and images for: Androgen Deprivation Therapy–Induced Muscle Loss and Fat Gain Predict Cardiovascular Events in Prostate Cancer Patients
Source: J Cachexia Sarcopenia Muscle. 2025 Jun 4;16(3):e13844. doi: 10.1002/jcsm.13844 (PMC12134783; doi:10.1002/jcsm.13844)

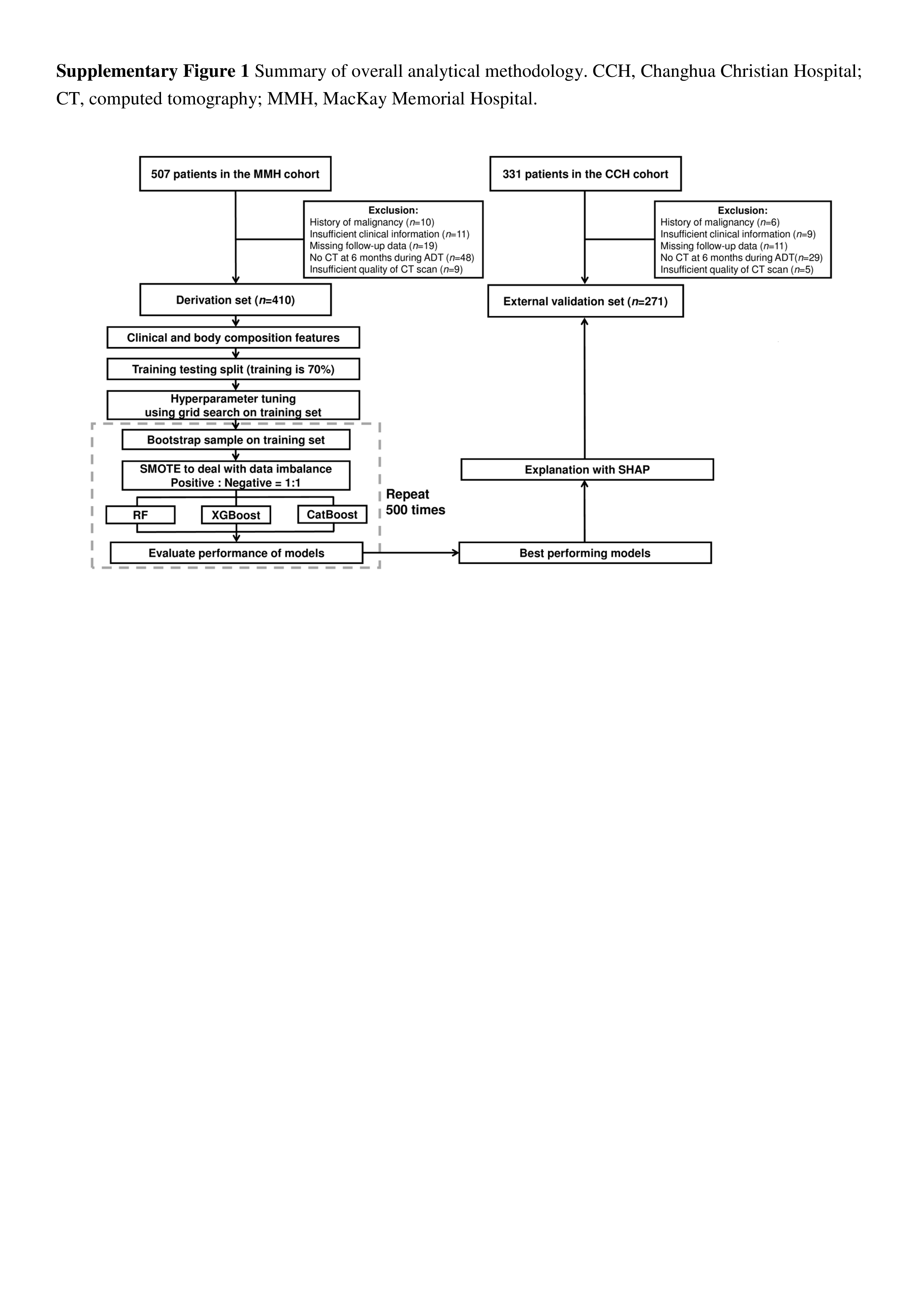

Supplement: Supplementary file 2 — Figure S1. Summary of overall analytical methodolody. CCH, Changhua Christian Hospital; CT, computed tomograpgy; MMH, MacKay Memorial Hospital. [file JCSM-16-e13844-s001.tif]

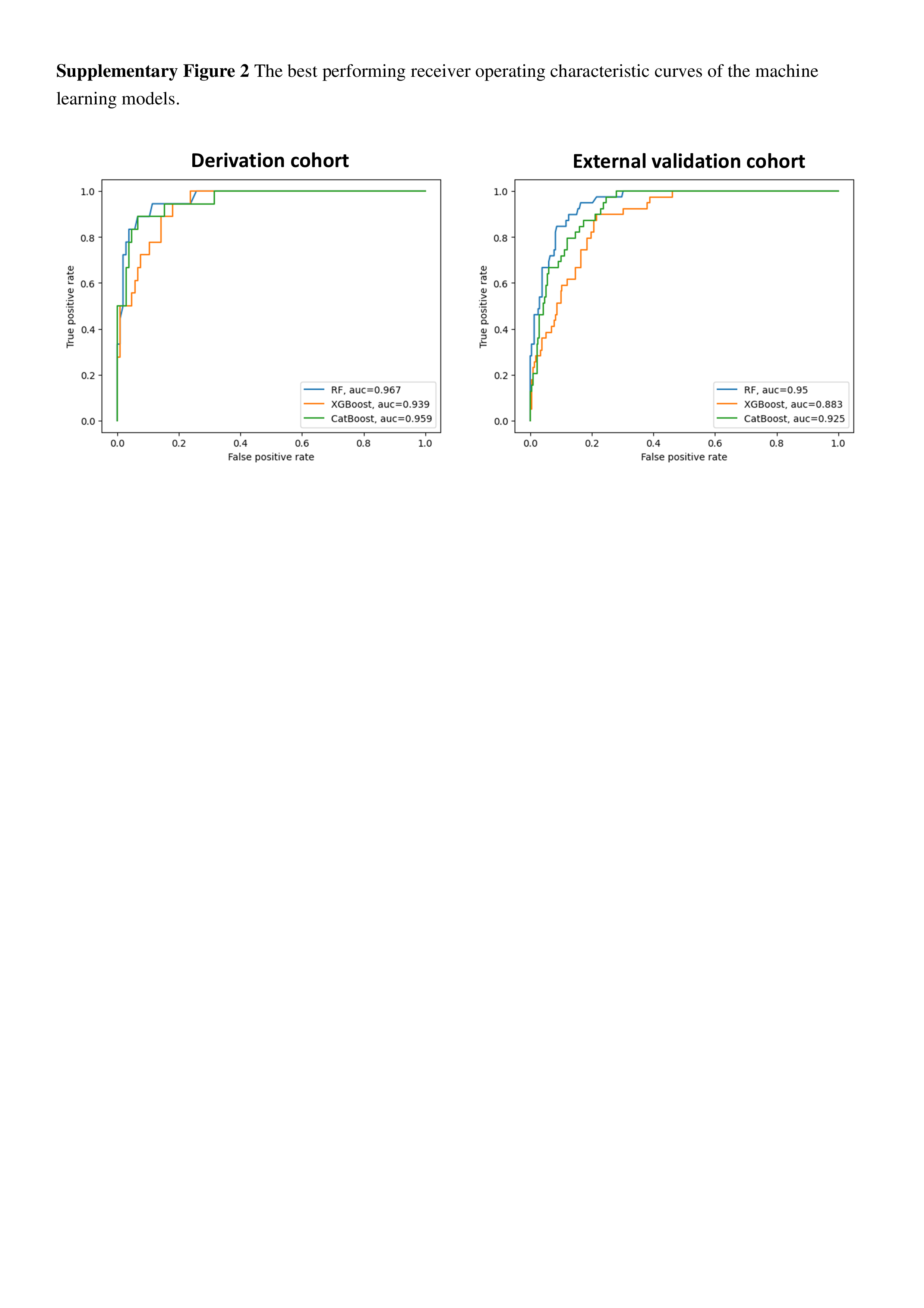

Supplement: Supplementary file 3 — Figure S2. The best performing receiver operating characteristic curves of the machine learning models. [file JCSM-16-e13844-s002.tif]
